# Studying novel high-pressure phases in laser-shock-affected silicon using poly: an algorithm for spot-wise phase identification

**DOI:** 10.1107/S1600576724011178

**Published:** 2025-02-01

**Authors:** Rasool Doostkam, Luca Gelisio, Aycan Yurtsever, Ludovic Rapp, Andrei V. Rode, Kenneth R. Beyerlein

**Affiliations:** a Institut National de la Recherche Scientifique – Énergie Matériaux Télécommunications Varennes Québec Canada; b European XFEL X-ray Free-Electron Laser Source Hamburg Germany; c Laser Physics Centre, Department of Quantum Science and Technology, Research School of Physics Australian National University Canberra Australian Capital Territory Australia; Tohoku University, Japan

**Keywords:** phase identification, electron diffraction, polymorphism, laser–matter interaction

## Abstract

Fast quenching dynamics in confined laser-induced microexplosions have been shown to lead to localized shockwaves that can create nanometre-scale domains in novel high-pressure crystalline phases. In the case of silicon, new silicon polymorphs such as bt8-Si and st12-Si have been recently observed, which are predicted to have bandgaps desirable for photovoltaic applications. Identification of these phases has been previously achieved by analysis of selected-area electron diffraction (SAED) patterns taken from laser-shock-affected areas. However, this analysis was complicated by pattern overlap from the many crystallites in the selected area, and many spots were found to agree with multiple potential phases. To overcome this ambiguity and enable the identification of the phase of Bragg spots observed in SAED patterns from polymorphic nanomaterials, we developed a new algorithm that we termed poly. This method is based on maximizing the magnitude and angular correlation between observed diffraction spots and those values derived from a known potential phase. We present the performance of this algorithm on simulated electron diffraction patterns as well as experimental SAED patterns measured from laser-shock-affected silicon samples. We find that the most abundant phases in the affected areas are t32-Si and t32*-Si and report on their relaxation into other high-pressure silicon phases over the course of 90 days after the laser-induced confined microexplosion.

## Abstract 2

This article describes a new phase identification algorithm called poly intended for spotty selected-area electron diffraction patterns collected from polymorphic nanomaterials. We have developed this new approach to determine the predominant high-pressure phases produced in laser-shock-affected regions of silicon. Applying this algorithm allowed us to reliably identify two new t32-Si and t32*-Si phases and indicate their relaxation time in other metastable high-pressure silicon phases.

## 1. Introduction

Our understanding of the structure and properties of materials under high pressure has been largely enabled by diamond anvil cell experiments, which have provided insights into several material phase transitions and metastable states (Sung, 1976 ▸; Evans *et al.*, 2007 ▸; McMillan, 2002 ▸). However, these devices are naturally limited by the strength of the diamond, resulting in a maximum achievable pressure of 640 GPa (Dubrovinsky *et al.*, 2012 ▸). Recently, a new approach was developed for exposing materials to a pressure level beyond the limit of the diamond anvil cell and preserving the high-pressure phases for further studies (Henderson *et al.*, 2021 ▸). The method is based on focusing high-energy ultrashort laser pulses inside the bulk of a transparent material to induce microexplosions in a confined geometry (Juodkazis *et al.*, 2006 ▸; Gamaly *et al.*, 2006 ▸, 2012 ▸). In this approach, the laser energy is deposited into the bulk of a transparent material on a very short timescale, <1 ps (1 ps = 10^−12^ s), faster than it can be dissipated by electronic heat conduction and electron–ion collision time. The deposition of only 1 µJ of laser pulse energy focused into a sub-micrometre-size focal volume immediately leads to an energy density of 1 MJ cm^−3^(1 MJ cm^−3^ = 1 TPa), which is higher than the strength of any material. The following fast plasma–solid transformation promotes the formation of metastable phases which can only be formed from the thermodynamically non-equilibrium high-entropy state of warm dense matter (Vailionis *et al.*, 2011 ▸; Rapp *et al.*, 2015 ▸). Following the rapid heating of the confined sub-micrometre volume, a highly localized shock wave is created that expands and dissipates its energy into the bulk. At the front of the shock wave pressures have been estimated to reach 10 TPa and temperatures above 10^5^ K (Juodkazis *et al.*, 2006 ▸; Gamaly *et al.*, 2006 ▸, 2012 ▸; Rapp *et al.*, 2015 ▸). This microexplosion is followed by highly non-equilibrium quenching conditions with ultrafast pressure release and ultra-high cooling rates (∼10^14^ K s^−1^), which give access to novel material states in local free energy minima far from equilibrium. Such novel phases remain trapped in a localized region in the pristine crystal which preserves them for later characterization. In this way, laser-shock-affected areas are created, and new high-pressure phases have been studied (Vailionis *et al.*, 2011 ▸; Rapp *et al.*, 2015 ▸; Juodkazis *et al.*, 2006 ▸). For example, super-dense aluminium has been formed from sapphire (Vailionis *et al.*, 2011 ▸), and phase transformations in olivine [(Fe, Mg)_2_SiO_4_] (Buividas *et al.*, 2014 ▸) have been reported using this approach.

Rapp *et al.* (2015 ▸) produced new phases in silicon by irradiating samples with 170 fs laser pulses of 790 nm wavelength. The laser was focused on a silicon surface buried under a transparent amorphous silicon dioxide (SiO_2_) layer, which acted to confine the microexplosion. Selected-area electron diffraction (SAED) measurements made on the affected area showed that the laser irradiation led to the formation of several new silicon metastable polymorphs. Laser fluences of 48 and 95 J cm^−2^ were found to result in the metastable phases of st12-Si and bt8-Si in the laser-shock-affected area, and there were indications of the potential existence of two more new structures, namely t32-Si and t32*-Si (Rapp *et al.*, 2015 ▸). The presence of these phases was also confirmed by Raman spectroscopy conducted on similarly prepared samples (Smillie *et al.*, 2020 ▸). This has been followed by theoretical studies of numerous other high-pressure phases (Wippermann *et al.*, 2016 ▸; Dmitrienko & Chizhikov, 2020 ▸). According to density functional theory simulations, the t32-Si phase can have a small quasi-direct bandgap of 1.28 eV which is useful in photovoltaic devices (Haberl *et al.*, 2016 ▸). Therefore, producing new phases of Si such as t32-Si and t32*-Si and distinguishing them from other high-pressure silicon structures is potentially important for future energy applications.

The challenge with performing quantitative phase analysis (QPA) of polymorphic nanomaterials using SAED measurements is that in many cases patterns contain overlapping diffraction patterns produced from nanocrystalline phase mixtures. The common approach to identifying phases using SAED patterns is only suitable for single crystals as it involves orienting crystals along a zone axis and comparing the measured pattern with that calculated from unit-cell information (Simbrunner *et al.*, 2021 ▸; Zaefferer, 2011 ▸; Lábár, 2005 ▸). Another common approach to performing QPA on nanocrystalline materials is to azimuthally average the SAED pattern and use software designed for powder diffraction to identify the observed peaks (Honglong *et al.*, 2013 ▸). However, this method reduces the information content of the pattern and is not able to distinguish the case when two phases have overlapping peak positions. Other approaches that have been reported for performing QPA on nanomaterials in a transmission electron microscope include studying the local Fourier transform of high-resolution images (Wang *et al.*, 2020 ▸), correlating with dark-field imaging to remove dominant phases (Rauch & Véron, 2019 ▸) and using a series of precession diffraction measurements (Moeck *et al.*, 2009 ▸; Eggeman, 2019 ▸; Midgley & Eggeman, 2015 ▸). However, each of these methods either has limitations on the sample crystallinity or requires specialized instrumentation. Meanwhile, SAED patterns contain rich information about the structure of the sample and can be collected using any transmission electron microscope. Furthermore, the ability to disentangle the information in overlapping diffraction patterns resulting from polycrystalline materials has been recently demonstrated by the development of multiple crystal indexing algorithms for X-ray diffraction measurements, such as triplet methods (Ohba *et al.*, 1981 ▸; Wright & Adams, 1992 ▸; Meng & Zuo, 2017 ▸), *Grainspotter* (Lauridsen *et al.*, 2001 ▸; Schmidt, 2014 ▸) and FELIX (Beyerlein *et al.*, 2017 ▸).

With this in mind, we have developed a new approach for phase identification of polymorphic materials from individual parallel-beam electron diffraction patterns like those routinely collected in SAED measurements. This leverages the correlations of angles between observed Bragg spots and those predicted from an assumed phase. It then scores this correlation in a spot-wise manner which allows for QPA of patterns from mixed-phase polycrystalline samples. This article starts with a description of the phase identification algorithm, referred to as poly. Then, the algorithm is tested on simulated diffraction patterns of the bt8-Si and st12-Si phases, as well as a mixture of patterns from both phases. Finally, the results of applying poly to the experimental data from laser-shock-affected areas of silicon are presented and discussed.

## 2. The poly phase identification algorithm

**Figure 1:**
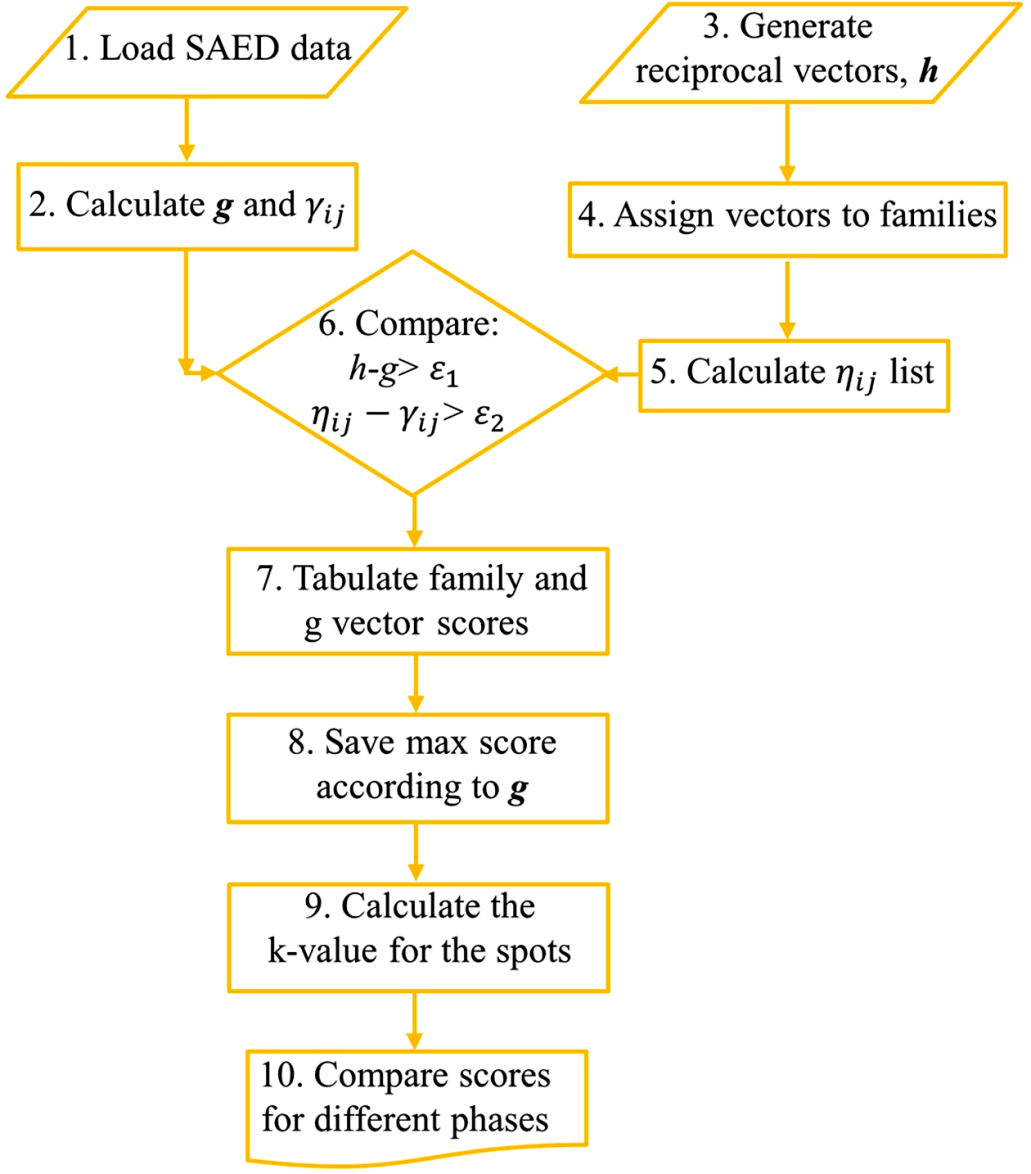
Flowchart of the poly phase identification algorithm.

Our algorithm calculates a score for each observed diffraction spot, reflecting its level of agreement with an assumed known phase. Its logic and organization follow the accumulation approach described by Morawiec (2020 ▸) for indexing and crystal orientation determination. A flowchart of the data processing steps of the algorithm is shown in Fig. 1 ▸. In step 1, the spots are found in the diffraction image using the approach detailed in Appendix *A*[App appa]. The center of the pattern is found, and each spot position is transformed into a vector in reciprocal space in step 2, using the relationship
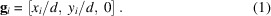
Here *x_i_* and *y_i_* are the coordinates of the spot center in the image, and *d* is the pixel-to-reciprocal-space conversion factor determined from detector distance calibration images. Then, a table of angles (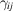) between all pairs of **g** vectors (**g**_*i*_, **g**_*j*_) is calculated.

Step 3 involves calculating the full set of reciprocal vectors, denoted by the variable **h**, from an assumed crystal structure, using the following equation:
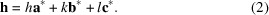
In this relationship, *h*, *k* and *l* are the Miller indices, and 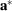, 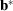 and 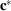 are reciprocal-space basis vectors defined as
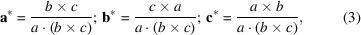
where **a**, **b** and **c** are unit-cell lattice vectors in real space. As a result, a list of all Bragg reflections having magnitudes up to a cut-off defined by the experimental SAED limit is generated. The forbidden reflections are removed from the list on the basis of the space group of the associated phase. In our case, it was not necessary to include spots arising from dynamical diffraction as we are studying small domains of high-pressure silicon phases in a deformed area of the silicon substrate. However, this framework allows dynamical diffraction spots to be included in this spot vector list generation step. Then, in step 4, the full list of reciprocal vectors is grouped according to families. A family is defined as a set of reciprocal vectors that are related by the symmetry operations of the Laue group irrespective of their magnitudes (Morawiec, 2020 ▸). For example, in the case of the phase t32-Si, which is tetragonal with 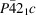 Laue group, only considering the vector [100] and space-group operators 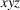 and 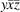, a family is generated, composed of the vectors [100], [010], [010], [100], and any parallel vectors like [*n*00] and [0*n*0], where *n* is an integer. In step 5, all angles between **h** vectors are calculated. These angles (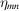) are indexed according to the associated families *m* and *n*. Then, duplicate angles with the same family indices are removed, resulting in a list of unique angles between pairs of families (Morawiec, 2020 ▸).

In step 6, we identify potential **h** vectors for an observed **g** by comparing their magnitudes following
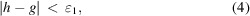
where 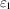 is a user-defined threshold. Then, the pairs of **g** vectors are selected and the list of angles between potential **h** vectors 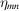 is compared with the angle 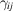 using the relationship
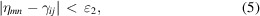
where 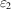 is a separately defined threshold for angles. We estimated these threshold parameters from the width of a Gaussian fit to the observed Bragg spots, as explained in Section 4.

In step 7, a vote table is generated with votes for **g** vectors organized in rows and those for families organized in columns. If equation (5) is satisfied, a vote is added to the table elements with indices (*i*, *m*), (*i*, *n*), (*j*, *m*) and (*j*, *n*). In this manner, votes are accumulated in the table considering all pairs of **g** vectors and potential families (Morawiec, 2020 ▸). Then, in step 8, the highest score for each **g** vector is selected and saved for later comparison. This also identifies the reflection families that correlate the most with the observed **g** vector.

To validate the calculated score, the algorithm conducts a null hypothesis test in step 9 to determine if the score is similar to that from a set of random spots without any crystallographic relationship. The parameters for this test were found by generating a set of diffraction patterns containing random spot positions and then using poly to determine the average (*M*) and standard deviation (Δ) of spot scores in the random data set for an assumed phase. These values were normalized by the number of spots in the pattern to allow for later comparison with an experimental score. Then, a *k*-score was calculated from the experimental scores following
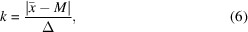
where 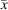 is the score of a spot from step 8 divided by the number of spots in the pattern. If the *k*-score of a spot is greater than 2, we reject the null hypothesis and consider the score further in the analysis. After applying this *k*-value filter to all scores, the final scores of a spot for different phases are compared.

This comparison is done in a spot-wise manner, allowing for the poly algorithm to identify diffraction patterns containing multiple crystals of different phases. In the next section, the performance of this algorithm is demonstrated on simulated patterns for multiple crystals in random orientations.

## 3. Phase identification of simulated SAED patterns

We simulated SAED patterns of the bt8-Si and st12-Si phases, which have distinct unit cells and space groups (Table 2) yet have many similar Bragg peak scattering vector magnitudes, which makes identification by existing approaches challenging. The pattern simulation proceeded by generating a list of allowed **h** vectors following equation (2) and considering the **h** vectors with magnitudes less than 6 nm^−1^. This value retains enough simulated Bragg spots for analysis, optimizing the operation time for poly. It also covers the same diffraction spots analyzed in the experimental data by Rapp *et al.* Then, a different orientation was created by rotating the **h** vectors according to a random set of Euler angles. The pattern was generated by finding all **h** vectors that satisfied the Ewald sphere condition, considering a wavelength of 0.0025 nm, corresponding to 200 keV electrons. An incident vector **S**_0_ with the form of [0, 0, 1/λ] was defined. Then, the magnitude of the vector **S** = **h** − **S**_0_ was compared with the Ewald sphere radius using
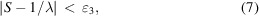
where 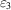 is a user-defined threshold assumed to be 2π/100 nm = 0.063 nm^−1^. This value was determined by comparing the number of spots in simulated patterns with that found in the later experimental measurements. The patterns including more than ten Bragg spots were kept for later analysis. The intensities of Bragg spots were not calculated because they are not considered in the poly algorithm.

**Figure 2:**
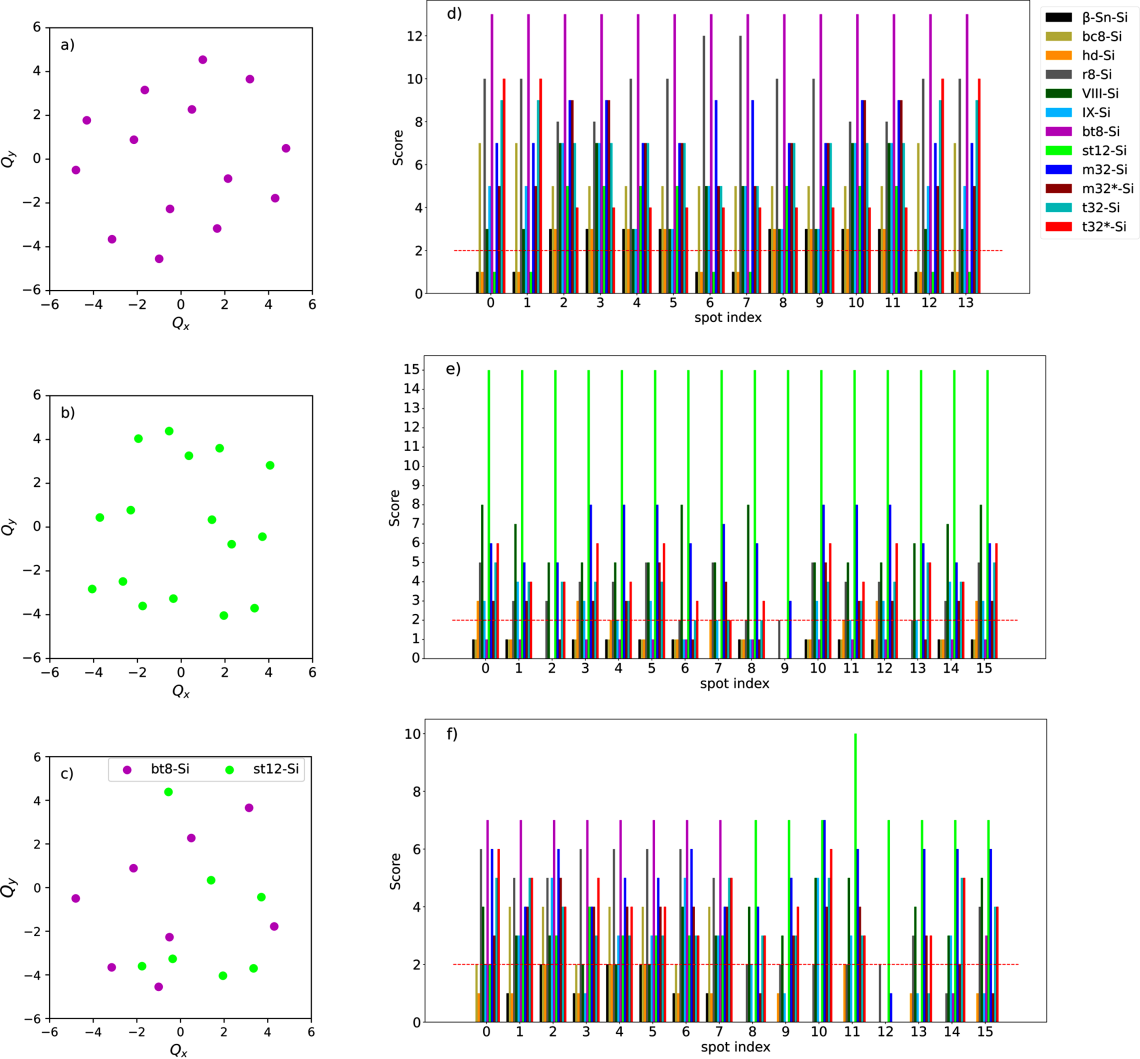
Simulated SAED patterns are shown for (*a*) a randomly oriented bt8-Si crystal and (*b*) a randomly oriented st12-Si crystal. (*c*) A mixed-phase SAED pattern was simulated using a selection of spots from those in (*a*) and (*b*). (*d*–*f*) Spot-wise similarity scores are compared assuming 12 Si phases in the shown diffraction patterns when analyzing the patterns (*a*–*c*) with the poly algorithm. The spots are colored according to the related phase with the highest scores.

Fig. 2 ▸(*a*) shows an example of the simulated SAED pattern for the bt8-Si phase. The poly algorithm described in the preceding section was first used to calculate similarity scores for the spots assuming the known phase, the results of which are shown as violet bars in Fig. 2 ▸(*d*). The assumed values for the thresholds in equations (4) and (5) were 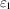 = 0.1 nm^−1^ and 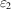 = 0.2°. Then the scores from the set of 11 candidate high-pressure phases of Si were considered, which are compared in the bar chart in Fig. 2 ▸(*d*). The lattice parameters and space groups assumed for each phase are listed in Table 2. The highest scores were used to color the spots in Fig. 2 ▸(*a*). As shown in the color bar, a higher score for bt8-Si is indicated by violet spots while a better agreement with st12-Si is shown in green.

In general, for a pattern of *N* spots, a spot can have *N* − 1 angles with others and therefore the highest score possible for a spot is *N* − 1. This value indicates that all angles with other spots agree with the assumed structure. For Fig. 2 ▸(*a*), 14 spots were simulated, and all spots obtained the theoretical maximum score of 13 when assuming the bt8-Si phase, which affirms the accuracy of the poly algorithm. When other phases were assumed, lower scores were found, which shows poly correctly identifies the phase of the spots.

An example pattern simulated for the st12-Si phase is shown in Fig. 2 ▸(*b*) and was analyzed using poly, assuming st12-Si and the other 11 phases of Si as before. The scores are plotted in Fig. 2 ▸(*e*). Again, in this case, the theoretical limit score of 15 was achieved when the st12-Si phase was assumed. The scores for st12-Si were found to be significantly higher than others, showing less ambiguity in the phase identification. Therefore, the poly algorithm also exhibits good efficiency in phase identification for the case of st12-Si.

To simulate a SAED pattern of a polycrystalline sample, we then randomly selected some spots from the two patterns in Fig. 2 ▸(*a*) and Fig. 2 ▸(*b*) and merged them into a single pattern in Fig. 2 ▸(*c*). This was then analyzed as before and the scores are shown in Fig. 2 ▸(*f*). Spots with indices from 0 to 7 were taken from the bt8-Si pattern, while those from 8 to 15 were taken from st12-Si. In Fig. 2 ▸(*f*), it is seen that each spot was correctly identified by receiving a higher relative score for the phase that matches the originally simulated pattern. Fig. 2 ▸(*c*) visually indicates the ability of the algorithm to identify the phase of the spots in this polycrystalline-like pattern.

These results show that the poly algorithm is capable of identifying a specific phase of Si from others. Furthermore, it works in a spot-wise manner and can correctly classify spots into different phases, which is applicable to polymorphic samples. One drawback of the poly algorithm is that it requires a predefined unit cell. However, we have found that slight deviations which may be caused by defects and strain can be accommodated by adjusting the threshold parameters ɛ_1_ and ɛ_2_. Finally, the poly algorithm will not work in the case of identifying spots from phases with similar lattice constants and space groups. This case is expected to lead to similar scores for those phases because of equivalent *d* spacing and angular relationships. Simulations like those presented can be used to test if multiple phases in the list of candidates are distinguishable by the algorithm. Still, in the event of a spot receiving similar high scores for multiple phases, we have found it is best to regard this as a subset of potential matches and compare this subset with how the scores are distributed for other spots in the pattern. As will be shown, this approach serves to significantly reduce the list of candidates and identify dominant phases in a SAED pattern.

## 4. Application of poly to SAED patterns of laser-shock-affected silicon

**Figure 3:**
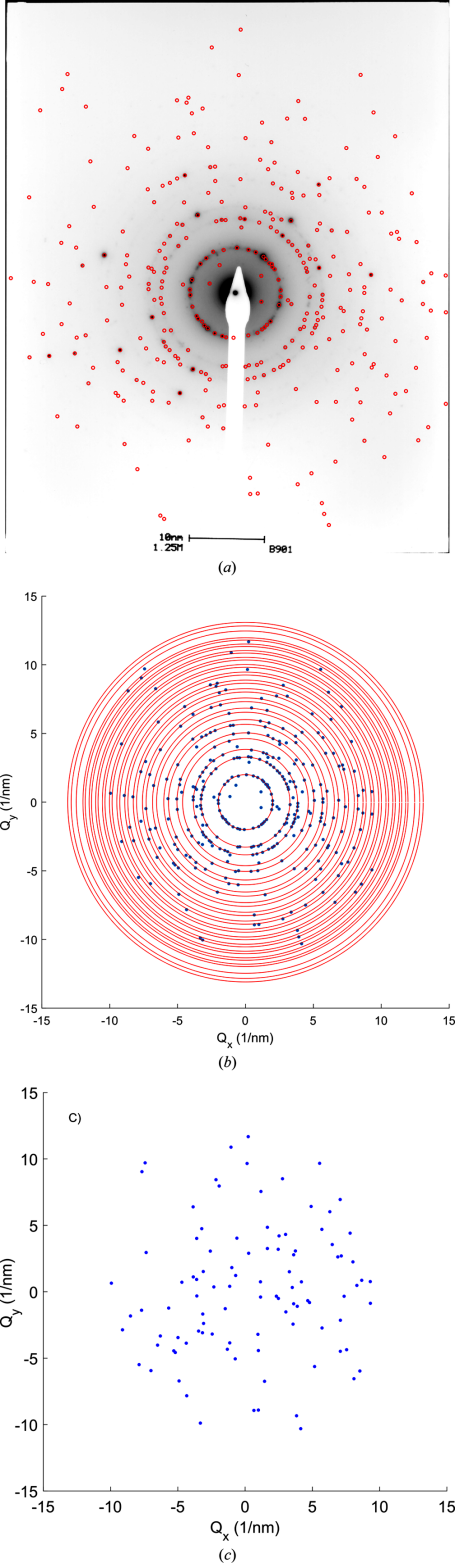
(*a*) Experimental SAED pattern of the Si sample that was irradiated by the laser. Detected spots are shown by red circles. (*b*) Detected spots are overlaid by the diffraction rings corresponding to the cubic phase of Si. (*c*) The spots remaining after filtering out the cubic phase of Si are shown.

We then applied the poly algorithm to measured SAED data of laser-shock-affected silicon samples created in the same conditions as reported by Rapp *et al.* (2015 ▸). Fig. 3 ▸ demonstrates the data preprocessing steps on the diffraction pattern labeled ‘b901’, which was also analyzed and presented as Fig. 3(*b*) in the original publication (Rapp *et al.*, 2015 ▸). First, the film-recorded diffraction pattern was digitized, and then the spots were found in the pattern using the algorithm detailed in Appendix *A*[App appa]. The locations of the found spots are shown as red circles overlaid with the diffraction pattern in Fig. 3 ▸(*a*). The center of the diffraction pattern was refined by using Friedel pairs in the found spot list and determining the intersection of lines connecting them. Then the detector distance was refined using a histogram of spot vector magnitudes and by identifying the first three major peaks as the first diffraction rings of the low-pressure silicon diamond cubic structure. This was used to transform the spot list into reciprocal-space coordinates, resulting in the **g** vectors plotted as the blue dots in Fig. 3 ▸(*b*).

**Figure 4:**
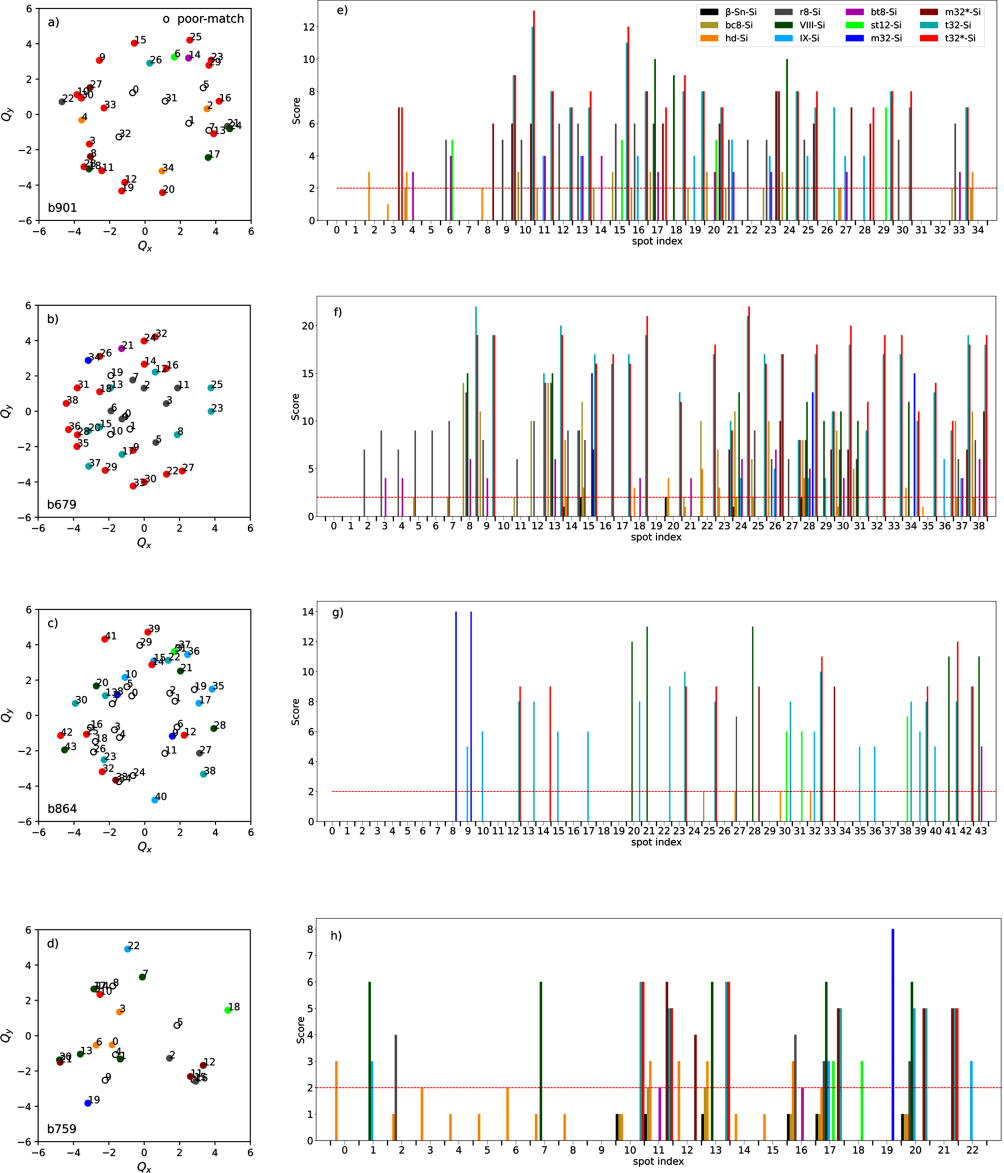
The experimental SAED patterns of the Si samples were extracted from Fig. 3 ▸(*c*) for pattern (*a*) and from other patterns in (*b*–*d*) considering *g* < 6 nm^−1^, and were analyzed by *g* and 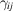 information using the poly algorithm. The spots in (*a*–*d*) are colored according to the highest scores in the (*e*–*h*) bars. The open black circles represent scores less than the similarity threshold for Si phases. In (*e*–*h*) spot-wise similarity scores assuming the Si phases in the analysis are compared. The threshold score of 2 is shown as a red dashed line.

To calculate the uncertainty in the locations of spots in the SAED pattern, we used a two-dimensional Gaussian fit for each spot so that the standard deviation of the fit provided the uncertainty in the *x* and *y* coordinates of the spots. Using these values, the uncertainties in the angles between pairs of spots were calculated. The values for the uncertainty in angles are in the order of 0.2°; therefore, we defined the threshold 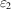 = 0.2° for the following reported analysis. After finding the location and uncertainty for each spot, we removed the spots that were found to have a scattering vector magnitude (*g*) that agreed with the cubic phase of Si. The result of filtering is shown in Fig. 3 ▸(*c*). Spots with *g* < 6 nm^−1^ were considered for further analysis. This spot list was then analyzed using poly, assuming the high-pressure phases described in Table 2. The results of the analysis for the pattern b901 are shown in Figs. 4 ▸(*a*) and 4 ▸(*e*) and summarized in Table 1 ▸.

We used poly to analyze a dataset of ten SAED patterns measured from different affected regions around voids created with incident laser fluences of either 48 or 95 J cm^−2^. Samples were prepared by a focused-ion beam (FIB) to produce thin cross sections of the voids suitable for transmission electron microscopy measurements. The time between the FIB sample preparation and the SAED measurement also varied between 34 and 94 days, which we will refer to as the ‘measurement delay’. Fig. 4 ▸ shows the results of analysis for four patterns that were found to have a significant number of spots that could be identified by the algorithm. The spots in the diffraction patterns are color coded in Figs. 4 ▸(*a*)–4 ▸(*d*) according to the phase that achieved the highest score for each spot, while the full score distributions are shown in the bar charts in Figs. 4 ▸(*e*)–4 ▸(*h*). The red line in the score distribution indicates a similarity score threshold that was introduced to identify spots that did not seem to have a significant agreement with any phase. If the spot received a score less than this threshold, it was designated as a poor match, and shown as open circles in the patterns. A list of experimental parameters and the number of spots matching each of the high-pressure silicon phases are given in Table 1 ▸.

The patterns measured with a SAED measurement delay of less than 50 days (b901 and b679) were found to predominantly have spots that had the highest similarity score to the t32-Si and t32*-Si phases. As seen in the score distributions, the poly algorithm attributed similar scores for each of these phases. This is because the unit-cell parameters are very similar (Table 2), making it hard for poly to distinguish between them. Interestingly, a higher number of t32-Si and t32*-Si spots were found in b679 than in b901. This seems to correlate with the laser fluence used for void creation, as the fluence for b679 was 95 J cm^−2^, while that for b901 was 48 J cm^−2^. Then, it appears that a higher laser fluence increases the fraction of the t32-Si phases created in the shock-affected region of the sample. Spots in these patterns assigned to other high-pressure phases were found in a significantly lower abundance. The phase r8-Si was found in both cases, while the hd-Si and VIII-Si phases were assigned to a few spots in the lower laser fluence measurement (b901).

The original analysis by Rapp *et al.* focused on the presence of the bt8-Si and st12-Si phases; however, the poly algorithm only found a few spots that matched with these phases. This difference is not surprising as the original analysis only considered the spot scattering vector magnitude and did not consider the angular correlations, as in the present approach. Furthermore, it focused on a few spots closest to the center of the pattern because the phase identification of the spots further from the center became increasingly ambiguous. Our present analysis does not exclude the presence of st12-Si and bt8-Si in the sample, as a few spots were indeed identified in the patterns and reasonable scores for these phases are found in the distributions shown in Figs. 4 ▸(*e*) and 4 ▸(*f*). Instead, our analysis suggests that the t32-Si and t32*-Si phases seem to be more abundant in the sample.

**Figure 5:**
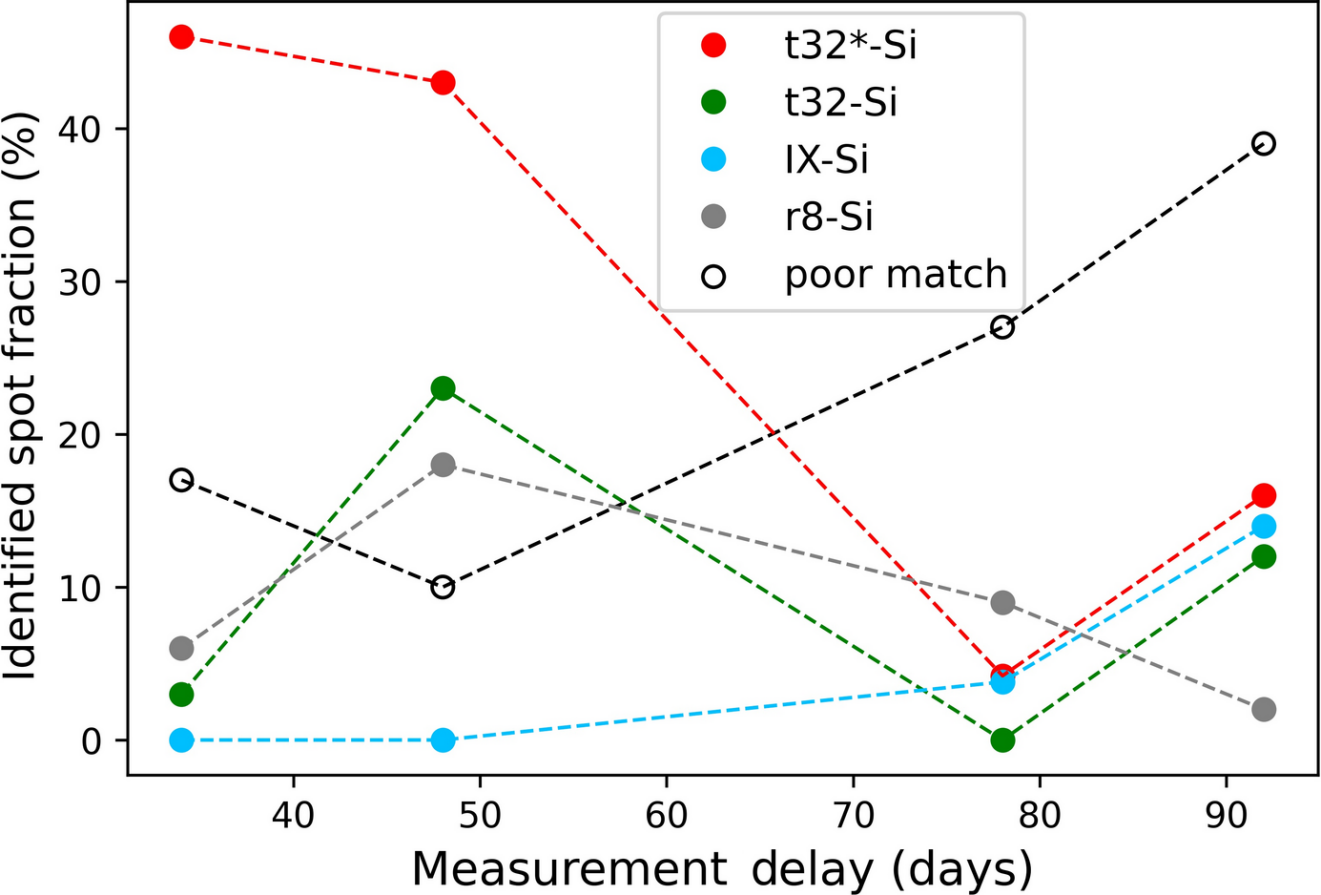
The fractions of spots attributed to the phases of silicon found to dominate the patterns shown in Fig. 4 ▸ are plotted as a function of the delay between the FIB preparation and SAED measurement of each pattern.

The different measurement delays of the patterns in the dataset have also allowed us to study the evolution of the stability of the high-pressure phase created in the affected region of the laser-shock volume. Creating the cross section and thinning the sample near the void removes residual stress and promotes the relaxation of the high-pressure phase to more low-pressure phases. To study this process, Fig. 5 ▸ contains a plot of the fraction of spots identified for each phase versus the measurement delay. It is observed that the number of spots in phases t32 and t32*-Si, shown in green and red, respectively, is decreasing over time. This reduction also occurs in phase r8-Si, albeit with a less steep decline. On the basis of these analyses, the relaxation time for phases t32/t32*- Si and r8-Si is estimated to be between 50 and 70 days. Conversely, the situation differs for phase IX-Si, with an increase in specified spots from 0% to 14%, suggesting that it is a by-product of the t32/t32*-Si phase relaxation.

It is noteworthy that, over time, the occurrence of spots in poor matches is also increasing. While the precise cause is unclear, this could be attributable to an unidentified phase or a lack of sufficient diffraction spots due to a small crystal size. Another potential explanation is an increased heterogeneity in the sample microstructure. In poly, the scores of spots depend on the number of spots (*N*) in a specific phase. If the variety of phases in a region increases, the scores decrease, and the probability of poor matches grows. To achieve more detailed insights in these cases, we plan to conduct nanobeam diffraction experiments to study the performance of poly on smaller affected volumes, which is expected to reduce the number of existing phases in the area and increase the relative scores.

## 5. Conclusion

We developed the poly algorithm to perform spot-wise QPA of nanoscale polymorphic materials. Diffraction patterns of bt8-Si and st12-Si were simulated to test the algorithm, and it was shown that the algorithm could find a reliable match between the detected diffraction spots in microexplosion experiments and the known high-pressure silicon phases. Moreover, we analyzed the experimental SAED patterns from laser-shock-affected Si samples, revealing the presence of novel high-pressure phases. The previous study by Rapp *et al.* showed that microexplosions in Si samples generated several tetragonal and monoclinic phases, but identifying them in a spot-wise manner using solely *d*-spacing information did not allow quantitative analysis. Our analysis with the poly algorithm, which uses angular and *d*-spacing information, has enabled the t32-Si and t32*-Si phases to be identified as the dominant phases in laser-shock-affected areas of silicon for the first time. In addition, analysis of a series of patterns with different measurement delays shows that these phases relax to other high-pressure phases in 50 days. Research is ongoing to test the limits of this approach to spot-wise phase identification on other material systems and identify the phases of the measured spots in new patterns by nanobeam measurements. Moreover, poly can be used on the position-sensitive diffraction patterns obtained from four-dimensional scanning transmission electron microscopy; however, in many cases, these patterns appear extremely diffuse. If the Bragg spots are sharp and well defined, poly can be helpful for phase identification.

## Figures and Tables

**Figure 6:**
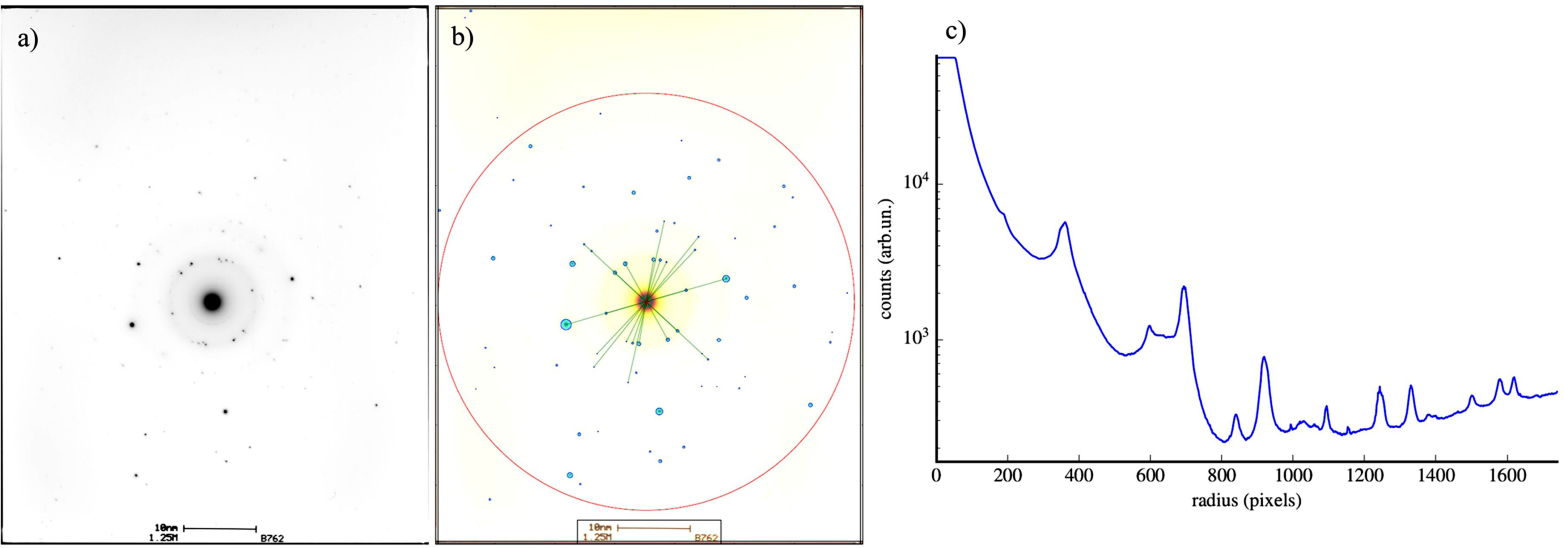
(*a*) A typical SAED pattern. (*b*) The algorithm found spots (light blue), pairs (connected by green lines) and the reciprocal-space origin (red cross). (*c*) The radial profile of the scattered intensity was obtained by employing the calculated reciprocal-space origin. The integrated region corresponds to the area enclosed by the red circle in (*b*).

**Table 1:** Experimental and analyzed data of four patterns of a laser-irradiated Si sample

	SAED pattern ID			
	b901	b679	b759	b864
Laser fluence (J cm^−2^)	48	95	95	95
Measurement delay (day)	34	48	78	92
No. of spots	35	39	23	44
hd-Si spots	3 (8%)	0	3 (13%)	0
r8-Si spots	2 (6%)	7 (18%)	2 (9%)	1 (2%)
VIII-Si spots	3 (8%)	0	5 (22%)	4 (9%)
IX-Si spots	0	0	1 (4%)	6 (14%)
bt8-Si spots	1 (3%)	1 (3%)	0	0
st12-Si spots	1 (3%)	0	1 (4%)	1 (2%)
m32-Si spots	0	1 (3%)	1 (4%)	2 (4%)
m32*-Si spots	2 (6%)	0	3 (13%)	1 (2%)
t32-Si spots	1 (3%)	9 (23%)	0	5 (12%)
t32*-Si spots	16 (46%)	17 (43%)	1 (4%)	7 (16%)
Poor match	6 (17%)	4 (10%)	6 (27%)	17 (39%)

**Table 2:** The unit-cell lattice parameters and Laue group of the silicon high-pressure phases considered

Phase	*a* (Å)	*b* (Å)	*c* (Å)	α (°)	β (°)	γ (°)	Laue group	Crystal family
β-Sn-Si	4.680	4.680	2.580	90	90	90	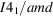	Tetragonal
bc8-Si	6.658	6.658	6.658	90	90	90	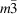	Cubic
hd-Si	3.850	3.850	6.364	90	90	120	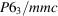	Hexagonal
r8-Si	5.650	5.650	5.650	110	110	110	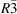	Trigonal
VIII-Si	8.627	8.627	7.500	90	90	90	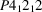	Tetragonal
IX-Si	7.482	7.482	3.856	90	90	90	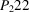	Tetragonal
bt8-Si	6.648	6.648	6.461	90	90	90	4/*m*	Tetragonal
st12-Si	5.650	5.650	6.764	90	90	90	4/*mmm*	Tetragonal
m32-Si	5.763	11.039	9.321	90	79.98	90	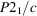	Monoclinic
m32*-Si	9.390	13.305	6.626	90	134.81	90	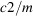	Monoclinic
t32-Si	9.408	9.408	6.646	90	90	90	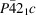	Tetragonal
t32*-Si	9.403	9.403	6.655	90	90	90	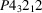	Tetragonal

**Table 3:** Weighting scheme Different features contributing to the definition of the weighting scheme are obtained as the sum of the value in each row of the second column.

	Attribute	Definition
1	Pairs lying on parallel lines	*w* = 1
2	Integrated intensity similarity	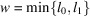 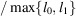
3	Average circularity	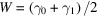

## Appendix A

### A1. Unit-cell parameters of silicon phases

These are given in Table 2 ▸.

### A2. The spot-finder algorithm

Here we describe the spot-finder algorithm that was used to process the measured SAED images presented in Section 4 of the article. It served to identify the diffraction spots and measure attributes like their size, circularity and location. After loading a SAED image, like that shown in Fig. 6 ▸(*a*), preprocessing was performed using routines from the *Open Source Computer Vision Library* (*OpenCV*) (Itseez, 2015 ▸). To reduce sharp intensity fluctuations, a Gaussian filter operation was performed using the Gaussian Blur function of *OpenCV* with a kernel size of 5 × 5 pixels and σ = 1.1. The region defined by the black rectangles in Fig. 6 ▸(*b*) was excluded from the image.

Then, intensity thresholding was performed to create a binary image that was used to identify candidate spots. Local adaptive thresholding was applied to overcome the large change in background level found in the image as a function of distance from the center. As seen in Fig. 6 ▸(*c*), the background was found to change by more than an order of magnitude across the image. To account for this, the *OpenCV* adaptive threshold function was used assuming a threshold value of μ − 10, where μ is the mean intensity over a given region of 751 × 751 pixels. Then, to remove thin-intensity clusters, morphological erosion was performed with a kernel size of 5 × 5 pixels.

The next step was spot finding, which started with processing the binary image. A spot was defined as a group of bright connected pixels satisfying the following size and shape criteria: the size condition was defined as a minimum of 10 pixels and a maximum of half the number of pixels defining the image width, while for the shape the number of pixels along one direction must be less than twice the number along the other. For pixels at the coordinates  with the intensity values (*I_i_*), the integrated intensity (*I*) and the center of the intensity () were defined as follows:

The summations run over each pixel in the cluster. The moment of inertia () relative to the center of intensity and the circularity () of the data were, respectively, expressed as

where the circularity is related to the similarity of the intensity distribution and a circle of radius  and *C* refers to the center of intensity.

After the spots had been found in the SAED pattern, the last step was the identification of the reciprocal-space origin. The first guess of the center position was found by calculating the center of mass of the spots that were more than 10% away from the center of the image. The center of the diffraction pattern was then refined by looking for Friedel pairs in the image. Pairs were found by starting from a spot at **p***_i_* and then searching for a spot within a circle at  with a radius of 5 pixels. If a spot was found to fall within the projected circle, it was documented as a pair of the spot at **p**_*i*_.

The statistical weight of each pair was defined according to different considerations that are summarized in Table 3 ▸. If the spot belonged to more than one pair because of peak splitting, the pair was deleted. Otherwise, the weight was given by the sum of the value calculated according to each row in the table. Once the weighting scheme was defined, the reciprocal-space origin was located by performing a weighted average of each pair mean position, :

Finally, the spot list was created by defining the position with respect to , the integrated intensity and the circularity. Weighting schemes are detailed in Table 3 ▸.
